# Identification of Sorafenib as a Treatment for Type 1 Diabetes

**DOI:** 10.3389/fimmu.2022.740805

**Published:** 2022-02-15

**Authors:** Qin Zeng, Jianfeng Song, Dandan Wang, Xiaoxiao Sun, Yalun Xiao, Haowei Zhang, Yang Xiao, Zhiguang Zhou, Tuo Deng

**Affiliations:** ^1^National Clinical Research Center for Metabolic Diseases, and Department of Metabolism and Endocrinology, The Second Xiangya Hospital of Central South University, Changsha, China; ^2^Key Laboratory of Diabetes Immunology, Ministry of Education, and Metabolic Syndrome Research Center, The Second Xiangya Hospital of Central South University, Changsha, China; ^3^Clinical Immunology Center, The Second Xiangya Hospital of Central South University, Changsha, China

**Keywords:** type 1 diabetes, Th1, sorafenib, IL-12, NOD mice

## Abstract

Th1 cell activation is considered a key mediator of the pathogenesis of type 1 diabetes. Targeting IL-12-induced Th1 cell differentiation seems to be an effective way to block the development of type 1 diabetes. However, given the critical function of Th1 in the immune system, the potential side effects hinder the application of anti-Th1 therapy in the treatment of type 1 diabetes. To identify safe anti-Th1 treatment(s), we screened the FDA-approved tyrosine kinase inhibitor (TKI) drug library using an IL-12-induced Th1 differentiation cell model. We found that among the TKIs with little effect on T cell viability, sorafenib is the top contender for the inhibition of Th1 differentiation. Treatment of NOD mice with sorafenib significantly impeded the development of type 1 diabetes and ameliorated insulitis, which coincided with a specifically decreased accumulation of Th1 cell population in the pancreas but not in peripheral immune organs. Mechanistically, sorafenib indirectly inhibited janus kinase 2 (JAK2) activity and blocked IL-12-induced phosphorylations of JAK2 and signal transducer and activator of transcription 4 (STAT4). Since sorafenib is classified as an FDA-approved drug, it serves as a preliminary lead point for additional experimentation and may be a promising therapy for type 1 diabetes in humans.

## Introduction

Type 1 diabetes is an immune-mediated disease characterized by the impairment of insulin-producing pancreatic beta cells. Since the destruction of beta cells in type 1 diabetes is mainly mediated by pathogenic autoreactive T cells, targeting autoreactive T cells shows promise in treating type 1 diabetes. T cell-based immunotherapies, such as an anti-CD3 monoclonal antibody, CTLA4-Ig fusion protein and low-dose anti-thymocyte globulin, have emerged for the treatment of type 1 diabetes (1). These immunotherapies indeed have shown some benefits in human clinical trials, but their systemic immunosuppressive effects and transient efficiency limit the success of application. In the face of an increasing type 1 diabetes population, it is urgent to development more effective and safer T cell-based immunotherapies.

In the early stage of type 1 diabetes, antigen-presenting cells (APCs) present autoantigens such as preproinsulin (PPI), insulinoma-associated antigen 2 (I-A2), GAD, and zinc transporter (ZnT8) to CD4^+^ T cells (2). Meanwhile, APCs secrete IL-12 to trigger the differentiation of naïve CD4^+^ T cells into Th1 cells. The Th1 cells produce inflammatory cytokines including IFN-γ and IL-2 to activate CD8^+^ cytotoxic T lymphocytes and macrophages (3), and cooperate with them to attack pancreatic beta cells. Transfer of pathogenic Th1 cells into neonatal NOD mice initiated the onset of type 1 diabetes (4), while the depletion of Th1 cells by disrupting Tbet, a key transcription factor for Th1 cell polarization, effectively blocked insulitis and type 1 diabetes in NOD mice (5). Therefore, Th1 cell population is a critical mediator of type 1 diabetes pathogenesis. IL-12 is a key cytokine for the induction of Th1 cell differentiation, and IL-12-induced signal transducer and activator of transcription 4 (STAT4) activation is a paramount signaling pathway for Th1 cell differentiation (6). In NOD mice, daily administration of IL-12 increased type 1 diabetes incidence (7), while addition of an IL-12 antagonist decreased type 1 diabetes incidence (8, 9). In addition, disruption of STAT4 activation prevented the development of type 1 diabetes in NOD mice completely (10). In humans, *IL-12b* was identified as a susceptible type 1 diabetes gene (11). These studies suggest that IL-12 played an essential role, inducing Th1 cell differentiation in pathogenesis of type 1 diabetes. Therefore, targeting the IL-12/STAT4 axis to suppress Th1 cell may be an effective strategy for the treatment of type 1 diabetes.

Tyrosine kinases possess a catalytic subunit that transfers a phosphate group from ATP to one or more tyrosine residues in a protein, leading to conformational changes, affecting protein function. They are important mediators of the signaling transduction, regulating cellular activity such as cell division, differentiation, apoptosis, and metabolism, in response to external and internal stimuli (12, 13). Due to multiple functions of tyrosine kinases in a variety of biological processes, tyrosine kinase inhibitors (TKIs) have been developed for the treatment of many diseases, including cancer, autoimmune and inflammatory diseases, degenerative disease and infectious disease (14, 15). Up until now, 39 TKIs have been approved by FDA, and more are being tested in clinical trials (16). IL-12/STAT4 pathway is directly mediated by a tyrosine kinase, janus kinase 2 (JAK2) (17, 18), and may be indirectly regulated by tyrosine kinase 2 (TYK2) or other tyrosine kinases (19, 20). To identify new and safe Th1 cell differentiation inhibitors that can be used to treat type 1 diabetes, we screened the FDA-approved TKI drug sub-library in an *in-vitro* cell culture model of IL-12-induced Th1 cell differentiation. We found that sorafenib, a TKI mainly targeting Raf for the treatment of renal and liver cancers, strongly blocked IL-12-induced Th1 cell differentiation by inhibiting the phosphorylations of JAK2 and STAT4. Treatment with sorafenib in NOD mice inhibited Th1 cell accumulation in the pancreas, and prevented insulitis and diabetes. These results suggest that sorafenib may be a potential treatment for type 1 diabetes.

## Material and Methods

### Experimental Animals

Female NOD mice were purchased from the Model Animal Research Center of Nanjing University (Nanjing, China), 017581-B6.129S4-*Ifng*^tm3.1Lky^/J (GREAT) mice were purchased from the Jackson Laboratory (Bar Harbor, ME, USA), C57BL/6J mice were purchased from Slac Laboratory Animal Inc (Shanghai, China). All mice were housed in a specific-pathogen-free animal facility at Central South University (Changsha, China). 12-week-old female NOD mice were monitored for blood glucose twice per week and considered diabetic after two consecutive blood glucose readings were >250 mg/dl. All animal experiments were performed in accordance with the guidelines from the Department of Laboratory Animal Science, Central South University.

### Drugs and Treatments

Sorafenib tosylate (Nexavar) tablets were purchased from Byer (Leverkusen, Germany) and suspended in 1% sodium carboxymethyl cellulose for *in vivo* use. Eight-week-old female NOD mice were treated with 10 mg/kg body weight of sorafenib or vehicle once daily by oral gavage. Administration time varies by experiment. Cyclophosphamide (Cy, Sigma-Aldrich, St. Louis, MO) was diluted to 35 mg/ml with 0.9% sodium chloride solution before i.p. for the treatment of 12-week-old mice (300 mg/kg) to induce the onset of diabetes in female NOD mice. For the therapeutic treatment, diabetic female NOD mice were treated with 10 or 50 mg/kg body weight of sorafenib or vehicle once daily by oral gavage for three weeks.

### Histological Analysis of Insulitis

Pancreatic tissues from vehicle-treated or sorafenib-treated mice were fixed with 4% paraformaldehyde, embedded in paraffin, and 5-μm-thick sections were stained with hematoxylin and eosin. Insulitis scores were calculated by using the following scale: 0 = no infiltrate, 1 = peri-insulitis, 2 = moderate insulitis (<50%), 3 = severe insulitis (>50%).

### Isolation of Lymphocytes From Tissues and Intracellular Staining

The pancreas was dissected from vehicle and sorafenib-treated mice, and digested in 1.5 mg/ml collagenase IV (Worthington Biochemical, Lakewood, NJ) for 45 min at 37°C. Pancreatic lymph nodes (PLNs) and spleens were mechanically disrupted. Cells were filtered through a 70 μm filter to get single cell suspension. Lymphocytes from tissues were stimulated for 6 h with phorbol myristate acetate (50 ng/ml) and ionomycin (5 μM) in the presence of brefeldin A and monensin (Biolegend, San Diego, CA, USA). Cells were collected to stain with antibodies, and then fixed and permeabilized in cytofix/cytoperm buffer (BD Biosciences, San Jose, CA).

### Mouse T Cell Isolation and Th1 Differentiation

Naive CD4^+^ T cells from the spleens of 6-8 weeks old male GREAT mice were prepared using magnetic bead cell sorting (Miltenyi Biotec, Bergisch-Gladbach, Germany). The purity of CD4^+^CD44^low^CD62L^high^ T cell subset was validated by flow cytometry. These naïve CD4^+^ T cells were stimulated with 5 μg/ml pre-coated anti-CD3ϵ antibody (Biolegend) and 1 μg/ml soluble anti-CD28 antibody (Biolegend) in culture medium containing 10 ng/ml IL-12 (Peprotech, Cranbury, NJ, USA), 10 ng/ml IL-2 (Peprotech) and 10 μg/ml anti–IL-4 antibody (BD Biosciences) for 3 days. The culture medium was RPMI 1640 medium (plus 50 μM β-mercaptoethanol) supplemented with 10% FBS, 1% GlutaMax, and 1% Pen/Strep (Gibco, Shanghai, China). 1 × 10^5^ naive CD4^+^ T cells were cultured in 96-well plates with 100 μl culture medium per well.

### Human T Cell Isolation and Th1 Differentiation

Healthy volunteers were recruited from the medical students at the Second Xiangya Hospital and written informed consent was obtained. Human study was approved by the Ethics Committee of the Second Xiangya Hospital of Central South University. Peripheral blood mononuclear cells (PBMCs) from the peripheral blood of healthy volunteers were separated by Histopaque (Sigma-Aldrich, St. Louis, MO). Naive CD4^+^ T cells from PBMC were isolated by negative selection using Miltenyi beads according to the manufacturer’s instructions. The purity of CD4^+^CD45RA^+^CD45RO^-^ T cell subset was validated by flow cytometry. The purified naïve CD4^+^ T cells were stimulated with 5 μg/ml pre-coated anti-CD3ϵ antibody (Biolegend) and 2 μg/ml soluble anti-CD28 antibody (Biolegend) in culture medium containing 10 ng/ml IL-12 (Peprotech, Cranbury, NJ, USA), 10 ng/ml IL-2 (Peprotech) and 10 μg/ml anti–IL-4 antibody (eBioscience) for 5 days. The culture medium was RPMI 1640 medium supplemented with 10% FBS, 1% GlutaMax, and 1% Pen/Strep (Gibco, Shanghai, China). 1 × 10^5^ naive CD4^+^ T cells were cultured in 96-well plates with 100 μl culture medium per well. The culture medium was refreshed on day 3.

### Drug Screen

The FDA-approved TKI drug library was purchased from Selleck Chemicals (Houston, TX, USA), and was dissolved in DMSO or water to get a 10 mM concentration for screening. Naive CD4^+^ T cells were prepared from the spleens of 8-week-old male GREAT mice, 1×10^5^ Th0 cells/well were plated into 96-well plates and cultured for 72 h. Th0 cells were cultured with Th1 differentiation medium containing 10 μM of individual FDA-approved TKI drug, and the IFN-γ–expressing Th1 cells were defined by detection of YFP expression.

### Cell Viability Assay

Cell viability of Th0 cells was performed using standard trypan blue exclusion method and ATP chemiluminescence assay. After isolation of naïve CD4^+^ T cells (Th0) cells, 5×10^4^ Th0 cells/well were plated into 96-well plates and cultured for 48 h. Viable cells were assessed by trypan blue staining. Similarly, 1×10^3^ Th0 cells/well were plated into opaque 96-well plates and cultured for 48 h, and then detected by using the CellTiter-Lumi™ luminescent cell viability assay kit (Beyotime, Beijing, China), the absorbance of 570 nm was measured using a microplate reader (Enspire Multimode Plate Reader, Perkin Elmer).

### *E. coli*-Infection Mouse Model

Eight weeks old male C57BL/6J mice were treated with 10 mg/kg body weight of sorafenib or vehicle once daily by oral gavage. Two days after sorafenib treatment, C57BL/6J mice were injected intraperitoneally with 0.2 ml of suspensions of *E. coli* (ATCC 8739) at 10^7^ CFU. Bacteria were harvested by centrifugation at 4000 rpm for 15 min and washed twice in PBS, and then diluted in PBS to obtain the desired concentration for infection experiments. To determine injection success, bacteria in peritoneal lavage fluid from the infected mice were collected at 24 hours after infection, and cultured overnight at 37°C on Luria-Bertani Agar plates.

### *In-Vitro* Kinase Assay

Enzyme activity was measured using mobility shift assay in duplicate in 384-well plates. Dilution series of ten different concentrations were diluted in DMSO. Sorafenib was diluted to 50X of the final desired highest inhibitor concentration in DMSO, 10 μl of the compound was mixed with 90 μl of 1x kinase buffer to each well of the 96-well plate for shaking 10 min, then the mixture was transferred to a 384-well plate in duplicates. After assay plate preparation, enzyme solution and carboxyfluorescein-labeled peptide and ATP were added into the assay plate in turn. Luminescence signal was measured on Caliper (Perkin Elmer).

### Real-Time Quantitative PCR

Total RNA was extracted from the pancreas using the Trizol reagent (Invitrogen, Life Technologies, NY) following manufacturer’s instructions. cDNA was synthesized using the cDNA Synthesis Kit (Thermo-Fisher Scientific, MA) and primers (Sangon Biotech Shanghai, China). RT-qPCR was performed using the FastStart Universal Master mix (Roche, Germany) in an ABI ViiA 7 Real-Time Polymerase Chain Reaction System. Relative gene expression levels were normalized to housing-keeping gene beta-actin. The following primers were used: mouse Tbet (Tbx21; forward: 5’-TTCAACCAGCACCAGACAG-3’, reverse: 5’-AGACCACATCCACAAACATCC-3’), mouse IFN-γ (Ifng; forward: 5’-CTTTGGACCCTCTGACTTGAG-3’, reverse: 5’-TCTTCCACATCTATGCCACTTG-3’), mouse IL-1β (forward: 5’-TGAAATGCCACCTTTTGACAG-3’, reverse: 5’-CACGGGAAAGACACAGGTAG-3’), mouse IL-6 (forward: 5’-TGGAAATGAGAAAAGAGTTGTGC-3’, reverse: 5’-CTCTGAAGGACTCTGGCTTTG-3’), mouse TNF-α (forward: 5’-CTTCTGTCTACTGAACTTCGGG-3’, reverse: 5’-TGTCTTTGAGATCCATGCCG-3’), mouse Bcl6 (forward: 5’-CCATACAAATGTGATCGCTGC-3’, reverse: 5’- GTAGGGCTTTTCTCCAGAGTG-3’), mouse Rorc (forward: 5’-GCACCAACCTCTTTTCACG-3’, reverse: 5’-ACGACTTCCATTGCTCCTG-3’), mouse Foxp3 (forward: 5’-ACCCAGGAAAGACAGCAAC-3’, reverse: 5’-CTTCTCCTTTTCCAGCTCCAG-3’), mouse Gata3 (forward: 5’-CTTATCAAGCCCAAGCGAAG-3’, reverse: 5’-TCGATTTGCTAGACATCTTCCG-3’), mouse IL-21 (forward: 5’-ATCATTGACCTCGTGGCC-3’, reverse: 5’-CTTCGGGTCCTATGTGTTCTAG-3’), mouse Stat4 (forward: 5’-CTGGGAGTAAAGGAAACGAGG-3’, reverse: 5’-GCTGGTCTCTAGGTTAATGGTG-3’), mouse Jak2 (forward: 5’-TGAGGCCACCAGTAAAAGACA-3’, reverse: 5’-TTTTGCCAGACAAGAGTGATG-3’), mouse beta-actin (forward: 5’-ACCGTGAAAAGATGACCCAG-3’, reverse: 5’-GAGCATAGCCCTCGTAGATG-3’).

### *In-Vitro* Phosphorylation Assay

The purified naive CD4^+^ T cells were cultured in RPMI 1640 with 10% FBS and 1% Pen/Strep. 2×10^6^ Th0 cells/well were pre-treated with gradient concentrations of sorafenib for 3 h and then stimulated with 10 ng/ml IL-12 for 5 min to induce the phosphorylations of JAK2 and STAT4. Finally, cell extracts were collected for western blots analysis.

### Western Blots Analysis

Total protein was extracted using RIPA buffer containing a cocktail of proteinase and phosphatase inhibitors (Sigma-Aldrich). Protein concentration was measured using BCA kit (Thermo Scientific, Pierce) and 30 μg/lane of total protein was loaded onto 10% polyacrylamide gels. The membranes were first incubated with following anti-phosphotyrosine antibodies at 4°C overnight: anti-pJAK2 (Cell Signaling Technology, Cat#3771, dilute 1:1000) and anti-pSTAT4 (Santa Cruz, Cat#sc-28296, dilute 1:100). The membranes were subsequently stripped using a solution containing 100 mM β-mercaptoethanol, 2% SDS, and 62.5 mM Tris-HCl (pH 7.6) at 50°C for 30 min, and re-incubated with anti-JAK2 (Cell Signaling Technology, Cat#3230, dilute 1:1000) or anti-STAT4 (Cell Signaling Technology, Cat#2653, dilute 1:1000) antibodies at 4°C overnight.

### Flow Cytometry

Cells were stained with fluorochrome-conjugated mAbs and analyzed on CYTEK northern lights NL-3000 or BD FACS Canto II. For surface markers detection, cells were incubated with FcR blocking antibody (Biolegend, Cat#101302 and 422302) for 15 min at room temperature, and stained with antibodies against surface markers for 30 min at 4°C in the dark. Stained cells were washed in PBS and then incubated with 7-AAD viability staining solution (Biolegend, Cat#420404) for 5 min at room temperature. For intracellular staining, cells were incubated with Zombie NIR (Biolegend, Cat#423106) for 15 min at room temperature, and then blocked with CD16/32 antibody and stained with fluorochrome-labeled mAbs against cell-surface antigens for 30 min at 4°C. Cells subsequently were fixed and permeabilized with foxp3/transcription factor staining buffer set (eBioscience, 00-5523-00) or cytofix/cytoperm buffer, and stained with fluorochrome-conjugated antibodies for an additional 30 minutes at 4°C in the dark. The following mAbs were used: CD45 (Biolegend, Cat#103108, 103132 and BD, Cat#562420), CD3e (Biolegend, Cat#100330, 100306, 100216), CD4 (Biolegend, Cat#100406, 100408, 100414, 980804 and eBioscience, Cat#11-0049-41), CD8a (Biolegend, Cat#100721), IFN-γ (Biolegend, Cat#505810, 505832 and 502512), IL-4 (Biolegend, Cat# 504123), IL-17A (Biolegend, Cat#506903), CD25 (Biolegend, Cat#102012 and eBioscience, Cat#56-0251-80), Foxp3 (Biolegend, Cat#126403 and eBioscience, Cat#17-57773-82), CD62L (Biolegend, Cat#104427), CD44 (Biolegend, Cat#103012), CD45RA (Biolegend, Cat#983002) and CD45RO (Biolegend, Cat#304230). Data were analyzed using FlowJo software.

### Statistical Analysis

Data are presented as means ± SEM. Statistical analysis was performed using SPSS Version 19.0 software (Chicago, IL, USA). Diabetes incidence was compared using log-rank test. Insulitis was analyzed with χ^2^ test. The other group differences were analyzed with 2-tailed Student’s t test.

## Results

### Sorafenib Inhibits IL-12-Induced Th1 Cell Differentiation

**Figure 1:**
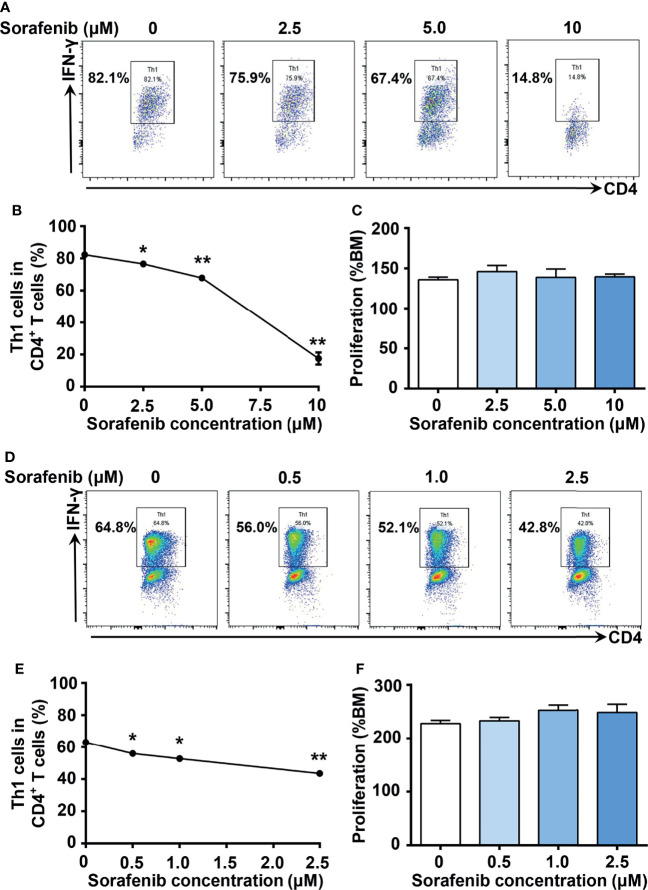
Sorafenib inhibits IL-12-induced Th1 cell differentiation in a dose-dependent manner in both mice and humans. **(A, B)** Flow cytometry analysis of Th1 cells in mouse naïve splenic CD4^+^ T cells stimulated with IL-12 in medium containing different concentrations of sorafenib as indicated. **(C)** CellTiter-Lumi assay to assess viable cells in the naïve splenic Th0 cells cultured in medium containing different concentrations of sorafenib as indicated. **(D, E)** Flow cytometry analysis of Th1 cells in human naïve PBMC CD4^+^ T cells stimulated with IL-12 in medium containing different concentrations of sorafenib as indicated. **(F)** CellTiter-Lumi assay to assess viable cells in the naïve PBMC Th0 cells cultured in medium containing different concentrations of sorafenib as indicated. BM, blank medium. Data are representative of 3 independent experiments. Data represent means ± s.e.m; ^*^P < 0.05 or ^**^P < 0.01 versus 0 μM by t-test.

To identify new inhibitors of Th1 cell differentiation, we utilized the ‘GREAT’ (IFN-γ reporter with endogenous polyA transcript) mice model (21), which allows analysis of IFN-γ–expressing cells through the detection of YFP expression. We screened a mini compound library including all TKI drugs approved by FDA between 2001 and 2019 (**Supplementary Figures 1A–C**). To exclude the TKIs with significant cytotoxicity to T cells, we assessed the toxic effects of TKIs on T cells first. We found that 10 TKIs have minimal cytotoxic effects on T cells at a concentration of 10 μM. Among them, sorafenib, an oral multikinase inhibitor with antitumor properties, was identified as the most potent inhibitor of IL-12-induced Th1 cell differentiation. As shown in **Figures 1A, B**, sorafenib inhibited IL-12-induced Th1 cell differentiation in a dose-dependent manner. The gating strategy and purity of sorted mouse naïve CD4^+^ T cells were shown in **Supplementary Figures 2A, B**. To confirm that the inhibitory effect of sorafenib on Th1 cell differentiation was not due to cytotoxicity, we treated naïve CD4^+^ T (Th0) cells with different doses of sorafenib for 2 days and examined the viability of naïve CD4^+^ T cells by performing an ATP chemiluminescence assay. We found that the number of viable cells did not differ by the treatment of 10 μM of sorafenib (**Figure 1C**), which strongly inhibited IL-12-induced Th1 cell differentiation. However, sorafenib was not able to inhibit IFN-γ production in Th1 cells at non-toxic doses (**Supplementary Figures 3A, B**). To determine whether sorafenib inhibits Th1 cell differentiation in humans, naïve CD4^+^ T cells were isolated from PBMC of healthy donors by cell sorting and subsequently were differentiated into Th1 cells by IL-12 stimulation. The gating strategy and purity of sorted naïve CD4^+^ T cells were shown in **Supplementary Figures 2C, D**. As shown in **Figures 1D–F**, sorafenib effectively inhibited IL-12-induced Th1 cell differentiation in human T cells without altering their viability. Collectively, our results indicate that sorafenib is a strong inhibitor of Th1 differentiation.

### Sorafenib Administration Prevents Development of Type 1 Diabetes

**Figure 2:**
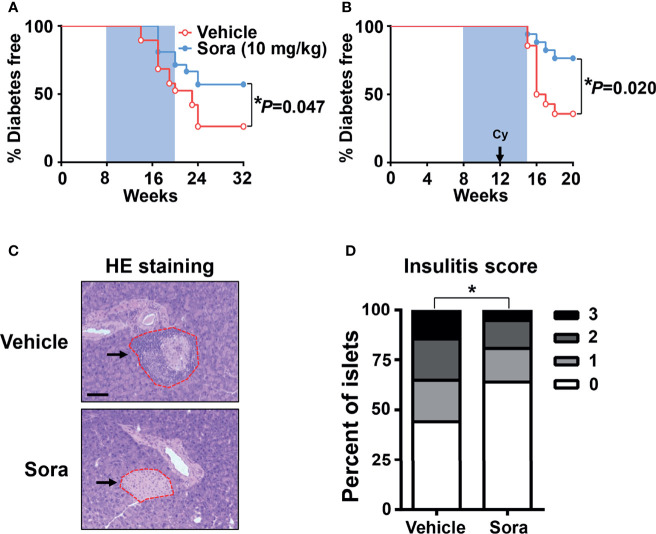
Sorafenib prevents the development of autoimmune diabetes in NOD mice. **(A)** Diabetes incidence of female NOD mice having been treated (gavage) daily with vehicle (n = 19) or sorafenib (10 mg/kg, n = 21) for 12 weeks (blue shaded area). **(B)** Diabetes incidence of female NOD mice having been treated daily with vehicle (n = 14) or sorafenib (10 mg/kg, n = 17) for 7 weeks. Cyclophosphamide (Cy, 300 mg/kg) was injected i.p., 4 weeks after beginning of sorafenib treatment. Statistical significance: ^*^P < 0.05 versus vehicle by long-rank test. **(C)** Representative images of HE staining for pancreatic sections from 14-week-old prediabetic NOD mice having been treated daily with sorafenib (10 mg/kg) or vehicle for 6 weeks, Cy (300 mg/kg) was injected i.p., 4 weeks after beginning of sorafenib treatment, the arrows point to the islet in pancreas. (scale bar indicates 100 μm). **(D)** Insulitis scores from 14-week-old prediabetic NOD mice, data are combined from two independent experiments. ^*^P < 0.05, χ^2^ test.

Type 1 diabetes is characterized by the activation of pathogenic autoreactive Th1 cells in the pancreas (3). To investigate whether sorafenib inhibits the development of type 1 diabetes, sorafenib was administered to NOD mice, a widely used type 1 diabetes mice model, once daily by oral gavage at a dose of 10 mg/kg body weight. Oral administration was initiated at 8 weeks of age and continued to 20 weeks of age. During the development of natural diabetes, vehicle-treated mice had a higher incidence (32%) than sorafenib-treated mice (19%) at 17 weeks of age, and the gap between two groups grew over time. By 32 weeks of age, the endpoint of this experiment, 43% of the mice treated with sorafenib were diabetic, as opposed to 74% of the vehicle-treated mice (**Figure 2A**). Next, we assessed the effects of sorafenib on cyclophosphamide (Cy)-induced type 1 diabetes. Cy treatment leads to a rapid onset of type 1 diabetes both in human and NOD mice through suppression of regulatory T cells (Tregs) (22–25). Our data showed that more than half of the vehicle-treated mice developed diabetes within 6 weeks after Cy injection, in contrast, only 24% of the sorafenib-treated mice developed diabetes during the same period (**Figure 2B**). Notably, the protective effects of sorafenib persisted and lasted for at least 5 weeks after cessation of oral administration (**Figure 2B**), suggesting that the short-term treatment had prolonged beneficial effects on the development of diabetes in this setting. Pancreatic islets were examined in vehicle-treated and sorafenib-treated prediabetic mice. After 2 weeks of Cy treatment, the mononuclear cell accumulation was less evident in the sorafenib-treated group when compared with the vehicle-treated group (**Figure 2C**), and mice treated with sorafenib showed a significantly lower insulitis score compared with the control mice (**Figure 2D**). Collectively, these results show that sorafenib impedes the development of type 1 diabetes and alleviates insulitis in NOD mice.

### Sorafenib Administration Diminishes Th1 Cells Accumulation in Pancreas but Not in Peripheral Immune Organs in NOD Mice

**Figure 3:**
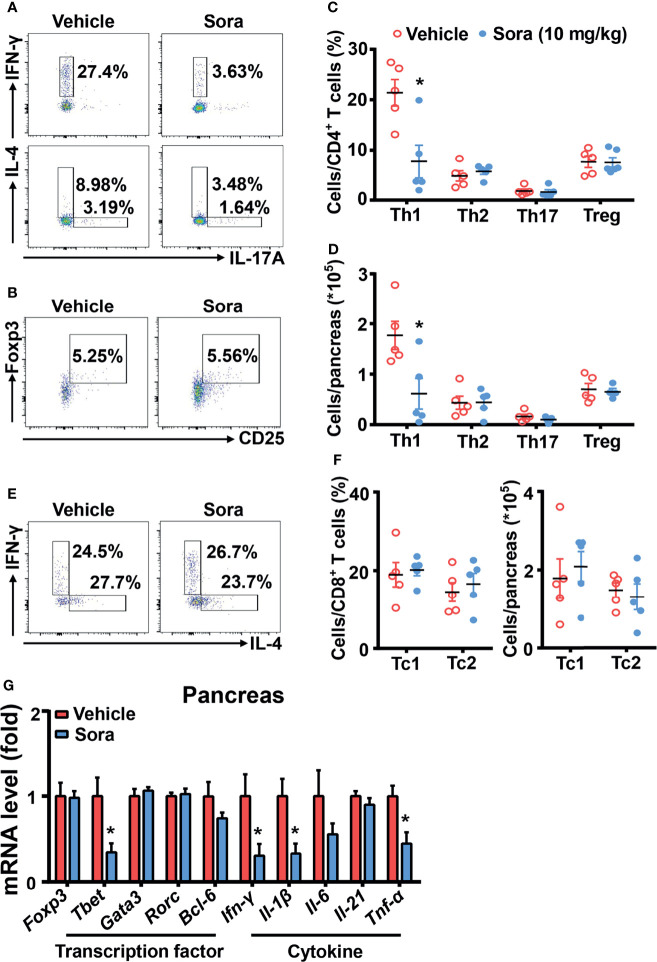
Sorafenib decreases inflammatory Th1 cells in the pancreas of prediabetic NOD mice. Flow cytometry analysis of the frequency and number **(A–D)** of Th1, Th2, Th17 cells and Tregs in pancreas from vehicle-treated or sorafenib-treated prediabetic NOD mice. Flow cytometry analysis of the frequency and number **(E, F)** of Tc1 and Tc2 cells in pancreas from vehicle-treated or sorafenib-treated prediabetic NOD mice. **(G)** RT-PCR analysis of transcription factors (Foxp3, Tbet, Gata3, Rorc and Bcl-6) and cytokines (Ifn-γ, Il-1β, Il-6, Il-21 and Tnf-α) in pancreas from vehicle-treated or sorafenib-treated prediabetic NOD mice. Sora, sorafenib. Data represent means ± s.e.m. and data were pooled from 2 or more independent experiments. n = 5-7 mice per group in each experiment. Statistical significance: ^*^P < 0.05 versus vehicle by t-test.

T cells play an important role in the development of type 1 diabetes (2). Our data (**Figure 1**) has shown sorafenib inhibits Th1 cell differentiation *in vitro*. To examine the effects of sorafenib on T cells *in vivo*, 8-week-old prediabetic NOD mice were treated with a therapeutic dose of sorafenib (10 mg/kg/day) for 6 weeks. These mice were injected with Cy 4 weeks after the beginning of the sorafenib treatment to promote accumulation of Th1 cells in pancreas. At the end of the sorafenib treatment, the T cells in the spleen, PLN and pancreas from the vehicle-treated or sorafenib-treated mice were analyzed with flow cytometry. Preventative treatment of sorafenib did not alter the CD4^+^ and CD8^+^ T cell ratio (data not shown) in spleen or PLNs, nor the CD4^+^ T cell subsets such as Th1, Th2, Th17 cell and Tregs populations (**Supplementary Figures 4A–C**). These data have indicated that prophylactic administration of sorafenib does not modify T cell subsets in peripheral immune organs. However, in the pancreas, the percentage and number of IFN-γ-producing Th1 cell were dramatically reduced by sorafenib administration, while the percentage and number of other CD4^+^ T cell subsets (Th2, Th17 and Treg cells) and CD8^+^ T cell subsets (Tc1 and Tc2 cells) were unchanged (**Figures 3A–F**). In addition, the mRNA levels of Th1 cell marker genes (*Tbet*) and proinflammatory cytokines (*IFN-γ*, *IL-1β*, *TNF-α*) were notably decreased in pancreas of sorafenib-treated mice when compared with vehicle-treated mice, but the mRNA levels of marker genes for Th2 (*Gata3*), Th17 (*Rorc*), Treg (*Foxp3*), and Tfh (*Bcl6*) cells and cytokine (*IL-21*) regulating Th1/Th17 cell differentiation were not different between the two groups (**Figure 3G**). To determine whether the inhibitory effect of sorafenib on Th1 differentiation is specific in pancreas, we investigated the effects of sorafenib on Th1 activation in mesenteric lymph node (MLN) and spleen of *E. coli*-infected C57BL/6J mice, an animal model of Th1 activation (26). The flow data showed that sorafenib effectively inhibited *E. coli*-induced Th1 cell activation in both MLN and spleen (**Supplementary Figures 5A–C**). Thus, it is likely that sorafenib, as an inhibitor of Th1 cell differentiation, induces Th1 deficiency and immunosuppression in the tissues accumulated with Th1 cells.

### Sorafenib Reverses Autoimmune Diabetes in NOD Mice

**Figure 4:**
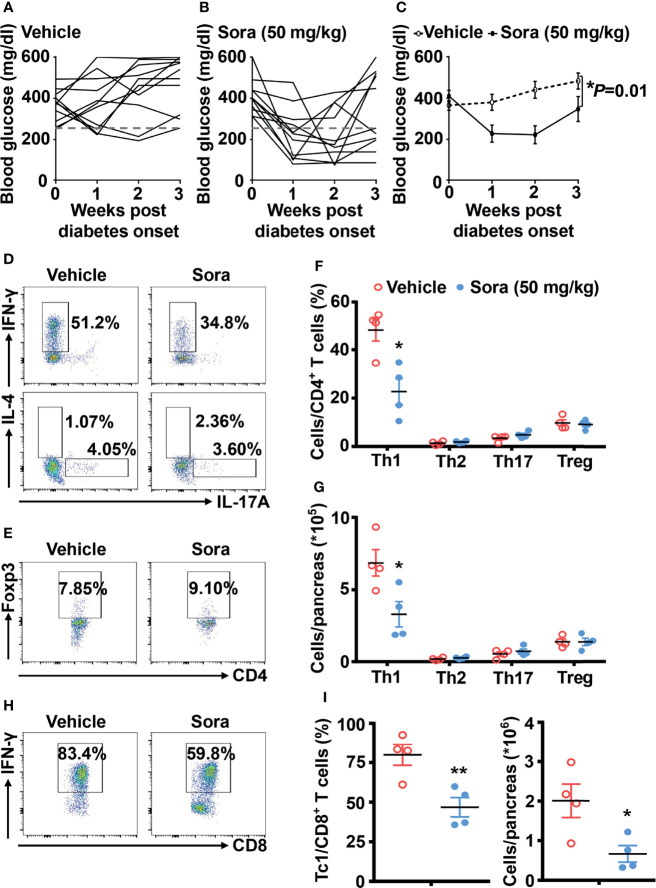
Sorafenib reverses autoimmune diabetes in NOD mice. The trend of blood glucose **(A–C)** from vehicle-treated (n = 11) and sorafenib-treated (50 mg/kg, n = 10) new-onset diabetic NOD mice (diagnosed by two consecutive readings of blood glucose>250 mg/dl). Flow cytometry analysis of the frequency and number **(D–G)** of Th1, Th2, Th17 cells and Tregs in pancreas from vehicle-treated or sorafenib-treated diabetic NOD mice. Flow cytometry analysis of the frequency and number **(H, I)** of Tc1 cells in pancreas from vehicle-treated or sorafenib-treated diabetic NOD mice. Sora, sorafenib. Data represent means ± s.e.m. N = 4 mice per group in each experiment. Statistical significance: ^*^P < 0.05, ^**^P < 0.01 versus vehicle by t-test.

In addition to defining its prevention role, we also tested whether sorafenib can reverse type 1 diabetes in NOD mice. Once diagnosed with diabetes, the mice were divided into three groups, including vehicle, low dose (10 mg/kg/day) and high dose (50 mg/kg/day) sorafenib groups. A low dose of sorafenib (10 mg/kg/day) did not reduce hyperglycemia in NOD mice with diabetes (data not shown). However, a high dose of sorafenib (50 mg/kg/day) significantly ameliorated hyperglycemia in diabetic NOD mice after one to three weeks of treatment (**Figures 4A–C**). The flow data showed that sorafenib did not alter the CD4^+^ T cell subsets such as Th1, Th2, Th17 cell and Tregs populations in the peripheral immune organs, PLN and spleen (**Supplementary Figures 6A–C**). In the pancreas, the frequency and number of Th2, Th17 and Treg cells were also unchanged, but Th1 and Tc1 cells were significantly decreased in the group of sorafenib-treated diabetic NOD mice (**Figures 4D–I**). These results suggest that sorafenib may create a less-inflammatory environment in pancreas of diabetic NOD mice, which leads to the functional recovery of islet beta cells and eventually the remission of autoimmune diabetes.

### Sorafenib Indirectly Inhibits the Activation of JAK2/STAT4 Pathway in IL-12-Induced Th1 Cell Differentiation

**Figure 5:**
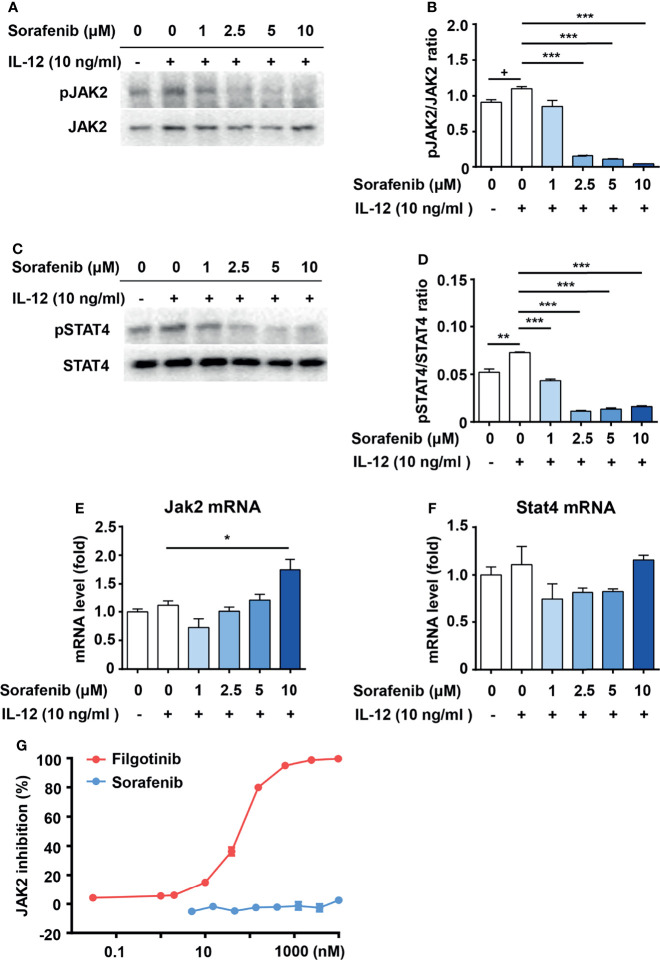
Sorafenib indirectly inhibits phosphorylations of JAK2 and STAT4. Immunoblots and quantitation of pJAK2 **(A, B)** and pSTAT4 **(C, D)** in naive CD4^+^ T cells, which were stimulated with IL-12 in medium containing different concentration sorafenib as indicated. Quantitation of phosphorylated JAK2 and STAT4 normalized to total JAK2 and STAT4. RT-PCR analysis of JAK2 and STAT4 in naive CD4^+^ T cells, which were stimulated with IL-12 in medium containing different concentration sorafenib **(E, F)** as indicated. **(G)** The inhibition ratio of filgotinib and sorafenib on JAK2 activity. Data are representative of 3 independent experiments. Data represent means ± s.e.m. Statistical significance: ^+^*P <* 0.1, ^*^P < 0.05, ^**^P < 0.01 or ^***^P < 0.001. T-test used for statistical analysis.

STAT4 is a transcription factor that mediates IL-12 induced Th1 cell differentiation (6). Disruption of STAT4 activation in NOD mice reduced Th1 type cytokine production and prevented the development of spontaneous type 1 diabetes (10). STAT4 protein is phosphorylated and activated by JAK2 (17, 18). To test whether JAK2 and STAT4 are involved in the inhibitory effects of sorafenib on Th1 cell differentiation, we measured the effects of sorafenib on JAK2 and STAT4 activity in IL-12 pretreated naive CD4^+^ T cells. As shown in **Figures 5A–F**, sorafenib had no effects on protein and mRNA levels of JAK2 and STAT4 at the concentration of 1-5 μM, but it strongly inhibited IL-12-induced phosphorylations of JAK2 and STAT4. Notably, the dose of sorafenib that inhibited JAK2 activation matched the dose that inhibited Th1 cell differentiation. Moreover, in order to determine whether sorafenib directly affects JAK2, we tested the effect of sorafenib on JAK2 protein. The results from *in-vitro* kinase assay showed that sorafenib had almost no inhibitory effects on JAK2 activity at a concentration from 1 μM to 10 μM (**Figure 5G**), indicating an indirect inhibitory effect of sorafenib on JAK2. Thus, these results suggested that sorafenib suppresses IL-12-induced Th1 cell differentiation by indirectly inhibiting JAK2/STAT4 axis activation.

## Discussion

To date, insulin therapy is still the standard treatment for type 1 diabetes. In most cases, blood glucose was well controlled by precise insulin administration, but hypoglycemia and complications are inevitable in some patients (27–29). With the unremitting efforts of researchers, islet or stem cell transplantation is explored as an emerging treatment for type 1 diabetes patients (30, 31). However, long-term immune rejection and islet autoantigen-specific T cells destroy the newly transplanted islet or stem cells, leading to failure of transplantation (32). Currently, a comprehensive cure for type 1 diabetes is not available. Since type 1 diabetes is basically an autoimmune disease, many have high expectations for controlling the excessive immune response in pancreas as a means for curing type 1 diabetes. Type 1 diabetes is mainly mediated by pathogenic autoreactive T cells, among which Th1 cells play a key role in the pathogenesis of type 1 diabetes (4, 5). Utilizing an *in-vitro* cell culture model, we found that sorafenib can effectively block IL-12-induced Th1 cell differentiation in a dose-dependent manner without affecting the viability of T cell in both mice and humans. This finding suggests that sorafenib may be able to fight type 1 diabetes, which is characterized by the over-expansion of Th1 cells in pancreas. Indeed, sorafenib administration prevented and reversed autoimmune type 1 diabetes in NOD mice by decreasing Th1 cells accumulation and expression of inflammatory cytokines in pancreas. Thus, sorafenib may have the potential to protect against type 1 diabetes by improving the immuno-environment in the pancreas.

The NOD mouse model is the most popular animal model of type 1 diabetes. However, it has some limitations for treatment assessment. In NOD mice, about 60%-80% female and 20%-30% male mice spontaneously develop type 1 diabetes, while the sexual discrepancy is not existed in human (33). The insulitis in diabetic NOD mice was more severe than that in patients with type 1 diabetes (34, 35). High numbers of CD4^+^ T cells and B cells predominate in NOD mice, while relatively small numbers of CD8^+^ T cells and macrophages were observed in patients with type 1 diabetes (36, 37). In addition, multiple susceptibility loci for type 1 diabetes, such as *Idd*3 and *Idd*5 loci, which have been found in human, are not directly shown to be dysregulated in NOD mice (33), highlighting the difference in the pathogenesis of type 1 diabetes between humans and NOD mice. Despite its limitations, the NOD mouse model is currently the sole animal model to test therapies for autoimmune diabetes *in vivo*.

Importantly, scattered clinical reports have provided evidence that sorafenib can affect blood glucose in malignancy patients. Sorafenib was reported to significantly reduce blood glucose in a patient with advanced diabetic hepatocellular carcinoma (38). In addition, a previous retrospective study showed that sorafenib can markedly reduce blood glucose levels in both diabetic and nondiabetic patients with cancers (39). These clinical studies suggest that sorafenib may have the ability to affect blood glucose in humans. Further studies are warranted to explore the therapeutic effect of sorafenib in patients with type 1 diabetes.

Since type 1 diabetes is an immune-mediated disease, the targeting of immune pathways is a continued focus on either preventing or reversing type 1 diabetes. Currently, there are no approved successful immunotherapies for type 1 diabetes, and past failures were mainly due to the various side effects from systemic immunosuppression (1). Given the comprehensive function of Th1 cells in the immune system, the potential side effects hinder the application of anti-Th1 therapy in the treatment of type 1 diabetes. Although sorafenib was identified as an inhibitor of Th1 differentiation, we believe it is quite a safe option for the treatment of type 1 diabetes. First, sorafenib has been approved by FDA for the treatment of multiple cancers for more than 15 years and has shown limited side effects (40, 41). Second, the dose of sorafenib (10 mg/kg/day for prevention and 50 mg/kg/day for remission) used in NOD mice to treat type 1 diabetes is lower or equal to the generally effective dose for the treatment of cancer (30-50 mg/kg/day) in animal models (42, 43), suggesting that the dose used to treat diabetes may be lower than the current clinical dose for cancer, which also means lower side effects. Third, our *in vivo* data showed that in NOD mice sorafenib decreased Th1 cells and inhibited inflammation specifically in the pancreas, but not in peripheral immune organs (**Figures 3A–C** and **Supplementary Figures 4A, B**), suggesting that sorafenib may not have significant effects on systemic immunity in certain condition. Notably, sorafenib repressed Th1 activation in spleens and MLNs of bacteria-infected mice. Since Th1 cells play a critical role in fighting bacterial infection, sorafenib may not be suitable to be used in patients with infectious diseases.

To date, the treatment of type 1 diabetes is still in a dilemma. Successful interventions at any stage of type 1 diabetes will be a breakthrough. Our study found that a low dose of sorafenib (10 mg/kg/day) has the ability to prevent (**Figures 2A, B**) but cannot reverse type 1 diabetes in NOD mice. However, high dose of sorafenib (50 mg/kg/day) treatment induces the remission of established type 1 diabetes (**Figures 4A–C**) in NOD mice, which may due to the suppression of Th1 and Tc1 cells in pancreas. This result indicates a therapeutic effect of sorafenib on autoimmune diabetes. We think sorafenib can be applied to treat type 1 diabetes, particularly under certain conditions. Sorafenib may be used for the treatment of latent autoimmune diabetes in adults (LADA), a subtype of type 1 diabetes which is characterized by a slower progression to insulin dependence. Patients with LADA were enriched with a subclass of GAD65 IgG1 autoantibodies, reflecting a dominant Th1 cell response to GAD65 in the pancreas (44). Moreover, compared with classic type 1 diabetes, LADA has a less functional deterioration of beta cells and a slower decline of C-peptide serum level (45). GAD autoantibody has been reported as a predictor of insulin requirement in LADA patients. The higher GAD autoantibody titers indicated the higher risk of early insulin dependence (46). Therefore, sorafenib may be particularly suitable for the treatment of LADA patients with higher GAD autoantibody titers. Another possible application area for sorafenib is the honeymoon phase of type 1 diabetes, a remission period where insulin usage can be sharply reduced or even withdrawn during the treatment of type 1 diabetes (47). Although the underlying mechanisms of honeymoon phase of type 1 diabetes are unclear, some clinical studies have indicated that the lower level of serum IFN-γ was associated with the remission of type 1 diabetes (48–50), suggesting that the sorafenib may be used to maintain and extend the honeymoon phase by reducing the accumulation of IFN-γ producing Th1 cells in the pancreas. These possible clinical applications of sorafenib are warranted to be explored in future studies.

Recent studies investigated the role of JAK-STAT signaling pathway in the pathogenesis of type 1 diabetes. The JAK1/JAK2 inhibitor AZD1480 reversed autoimmune diabetes in newly diagnosed mice by blocking the CD8^+^ T cell-mediated damage of beta cells (51). A case report showed that JAK1/JAK2 inhibitor ruxolitinib normalized blood-glucose levels and discontinued exogenous insulin in a patient with type 1 diabetes caused by STAT1 gain-of-function (52). A recent review summarized the roles of STAT (STAT1, STAT3 and STAT5b) monogenic mutations in type 1 diabetes. These mutations are associated with an increased susceptibility to type 1 diabetes due to the perturbation of Th17/Treg equilibrium (53). These findings suggest that JAK/STAT inhibitor is a potential therapy for type 1 diabetes. IL-12/JAK2/STAT4 signaling has been defined as an essential way for Th1 cell differentiation (17, 18). In our study, we found that sorafenib significantly suppressed IL-12-induced activations of JAK2 and STAT4 in a dose-dependent manner (**Figures 5A–D**), suggesting that sorafenib is a new JAK2 inhibitor. To further explore whether sorafenib directly affects JAK2, we examined the effect of sorafenib on JAK2 activity in an *in-vitro* kinase assay. The classic JAK2 inhibitor, filgotinib, almost completely inhibited JAK2 activity at concentrations as low as 0.6 μM (**Figure 5G**), indicating that the assay system worked very well. In this *in-vitro* kinase assay, sorafenib had little inhibitory effects on JAK2 activity at a concentration of 10 μM. However, this concentration of sorafenib was sufficient to inhibit the IL-12-induced phosphorylation of JAK2 in T cells. (**Figures 5A, B**). This result suggested that sorafenib cannot directly target JAK2, and may indirectly inhibit IL-12-induced JAK2 activation by targeting an unknown factor. Sorafenib is a multiple kinase inhibitor and directly targets Raf and several receptor tyrosine kinases, including vascular endothelial growth factor receptor (VEGFR), platelet derived growth factor receptor (PDGFR), FLT-3, c-Kit, p38 and RET (40). Although we cannot exclude the possibility that the known sorafenib targets mediate its inhibitory effects on JAK2 activation, it is more likely that sorafenib suppresses the IL-12-induced JAK2 activation through its unidentified target. Further studies are warranted to identify the new target of sorafenib.

## Conclusion

In summary, we identified sorafenib as an inhibitor of Th1 differentiation and an indirect inhibitor of JAK2 and found that sorafenib prevents and reverses type 1 diabetes in NOD mice by decreasing the accumulation of Th1 cells and the expression of inflammatory cytokines in pancreas. Our findings emphasize the importance of Th1 cells in the pathogenesis of type 1 diabetes and suggest that sorafenib may prove to be an important new therapy for clinical treatment in type 1 diabetes.

## Data Availability Statement

The original contributions presented in the study are included in the article/**Supplementary Material**. Further inquiries can be directed to the corresponding authors.

## Ethics Statement

The studies involving human participants were reviewed and approved by Ethics Committee of the Second Xiangya Hospital of Central South University. The patients/participants provided their written informed consent to participate in this study. The animal study was reviewed and approved by The Second Xiangya Hospital of Central South University ethics committee.

## Author Contributions

QZ and JS contributed to the study design, acquisition of data, analysis and interpretation of results, as well as drafting and revision of the manuscript. DW, XS, YLX, and HZ contributed to the acquisition of the data and revision of the manuscript. YX and ZZ contributed to the design of study, analysis of results and revision of the manuscript. TD contributed to the conception and design of the study, analysis of results and the revision of the manuscript. All authors gave their approval for the final manuscript to be published.

## Funding

This work was supported by the National Key R&D Program of China (2020YFA0803604), the National Natural Science Foundation of China (81770868 and 91742103), the National Science and Technology Major Project (2020ZX09201-28), the Science and Technology Innovation Program of Hunan Province (2020RC4009) and the Project of Innovation-Driven Plan of Central South University (2020CX015).

## Conflict of Interest

TD has filed for a provisional patent (pending) (CNIPA; publication number: 202010812329.3) regarding the use of sorafenib for the treatment of type 1 diabetes.

The remaining authors declare that the research was conducted in the absence of any commercial or financial relationships that could be construed as a potential conflict of interest.

## Publisher’s Note

All claims expressed in this article are solely those of the authors and do not necessarily represent those of their affiliated organizations, or those of the publisher, the editors and the reviewers. Any product that may be evaluated in this article, or claim that may be made by its manufacturer, is not guaranteed or endorsed by the publisher.

## Supplementary Material

Supplementary Figure 1 The effects of 34 FDA-approved TKIs on IL-12-induced Th1 cell differentiation. **(A)** Cell counts using trypan blue exclusion to assess viable cells and **(B, C)** flow cytometry analysis of Th1 cells in naïve splenic CD4+ T cells stimulated with IL-12 in medium containing 10 μM of individual FDA-approved TKI as indicated. Data represent means ± s.e.m; *P < 0.05 or **P < 0.01 versus vehicle by t-test.

Supplementary Figure 2 Gating strategy for flow cytometry analysis of T cells. **(A)** Gating of naïve CD4+ T cells in mouse splenocyte (upper panel); the purity of sorted naïve CD4+ T cells (down panel). **(B)** Gating of Th1 cells in mouse CD4+ T cells. **(C)** Gating of naïve CD4+ T cells in human PBMC (upper panel); the purity of sorted naïve CD4+ T cells (down panel). **(D)** Gating of Th1 cells in human CD4+ T cells. PBMC, peripheral blood mononuclear cells.

Supplementary Figure 3 Sorafenib does not alter IFN-γ expression in Th1 cell. **(A, B)** Flow cytometry analysis of IFN-γ + Th1 cells treated with different concentrations of sorafenib (μM) as indicated.

Supplementary Figure 4 Sorafenib does not alter T cell subsets in peripheral immune tissues of prediabetic NOD mice. Flow cytometry analysis of the frequency of Th1, Th2, and Th17 cells in PLN **(A)** and splenic **(B)** CD4+ T cells from vehicle-treated or sorafenib-treated NOD mice. **(C)** Flow cytometry analysis of the frequency of Tregs in PLN and splenic CD4+ T cells, respectively. Sora, sorafenib; PLN, pancreatic lymph nodes. Data represent means ± s.e.m. and data were pooled from 2 or more independent experiments. n = 5-7 mice per group in each experiment.

Supplementary Figure 5 Sorafenib decreases activate Th1 cells in MLN and spleen of E. coli-infected mice. **(A)** Image of E coli on Luria-Bertani Agar plates from vehicle-treated or sorafenib-treated C57BL/6J mice injected with PBS or E.coli (1*107 CFU). Flow cytometry analysis of the frequency of Th1 cells in MLN **(B)** and splenic **(C)** CD4+ T cells from the three groups mice as indicated. Sora, sorafenib; MLN, mesenteric lymph node. Data represent means ± s.e.m.. n = 4 mice per group in this experiment. Statistical significance: *P <0.05 or **P <0.01 versus vehicle by t-test.

Supplementary Figure 6 Sorafenib does not alter T-cell subsets in peripheral immune tissues of diabetic NOD mice. Flow cytometry analysis of the frequency of Th1, Th2, and Th17 cells in PLN **(A)** and splenic **(B)** CD4^+^ T cells from vehicle-treated or sorafenib-treated diabetic NOD mice. **(C)** Flow cytometry analysis of the frequency of Tregs in PLN and splenic CD4^+^ T cells, respectively. Sora, sorafenib; PLN, pancreatic lymph nodes. Data represent means ± s.e.m. n = 4 mice per group in each experiment.

## Abbreviations

APCs, antigen-presenting cells; Cy, Cyclophosphamide; I-A2, insulinoma-associated antigen 2; JAK2, janus kinase 2; LADA, latent autoimmune diabetes in adults; MLN, mesenteric lymph node; PBMCs, Peripheral blood mononuclear cells; PDGFR, platelet derived growth factor receptor; PLNs, pancreatic lymph nodes; PPI, preproinsulin; STAT4, signal transducer and activator of transcription 4; TKI, tyrosine kinase inhibitor; Tregs, regulatory T cells; TYK2, tyrosine kinase 2; VEGFR, vascular endothelial growth factor receptor; ZnT8, zinc transporter 8; Cy, cyclophosphamide; MLN, mesenteric lymph node; PMBC, peripheral blood mononuclear cells.
