# Supplementary figures and images for: Identification of Sorafenib as a Treatment for Type 1 Diabetes
Source: Front Immunol. 2022 Feb 15;13:740805. doi: 10.3389/fimmu.2022.740805 (PMC8886732; doi:10.3389/fimmu.2022.740805)

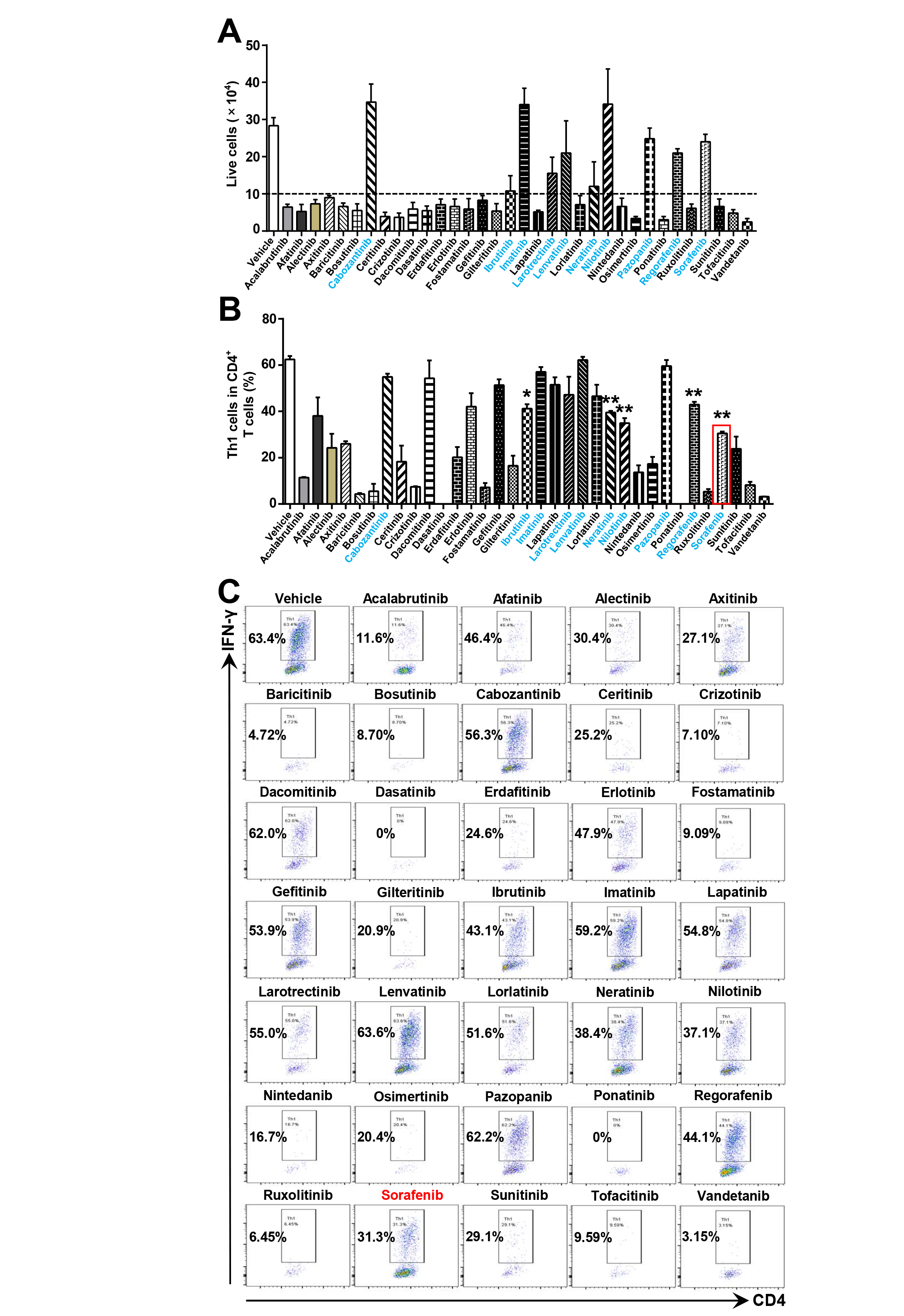

Supplement: Supplementary Figure 1 — The effects of 34 FDA-approved TKIs on IL-12-induced Th1 cell differentiation. (A) Cell counts using trypan blue exclusion to assess viable cells and (B, C) flow cytometry analysis of Th1 cells in naïve splenic CD4+ T cells stimulated with IL-12 in medium containing 10 μM of individual FDA-approved TKI as indicated. Data represent means ± s.e.m; *P < 0.05 or **P < 0.01 versus vehicle by t-test. [file Image_1.tif]

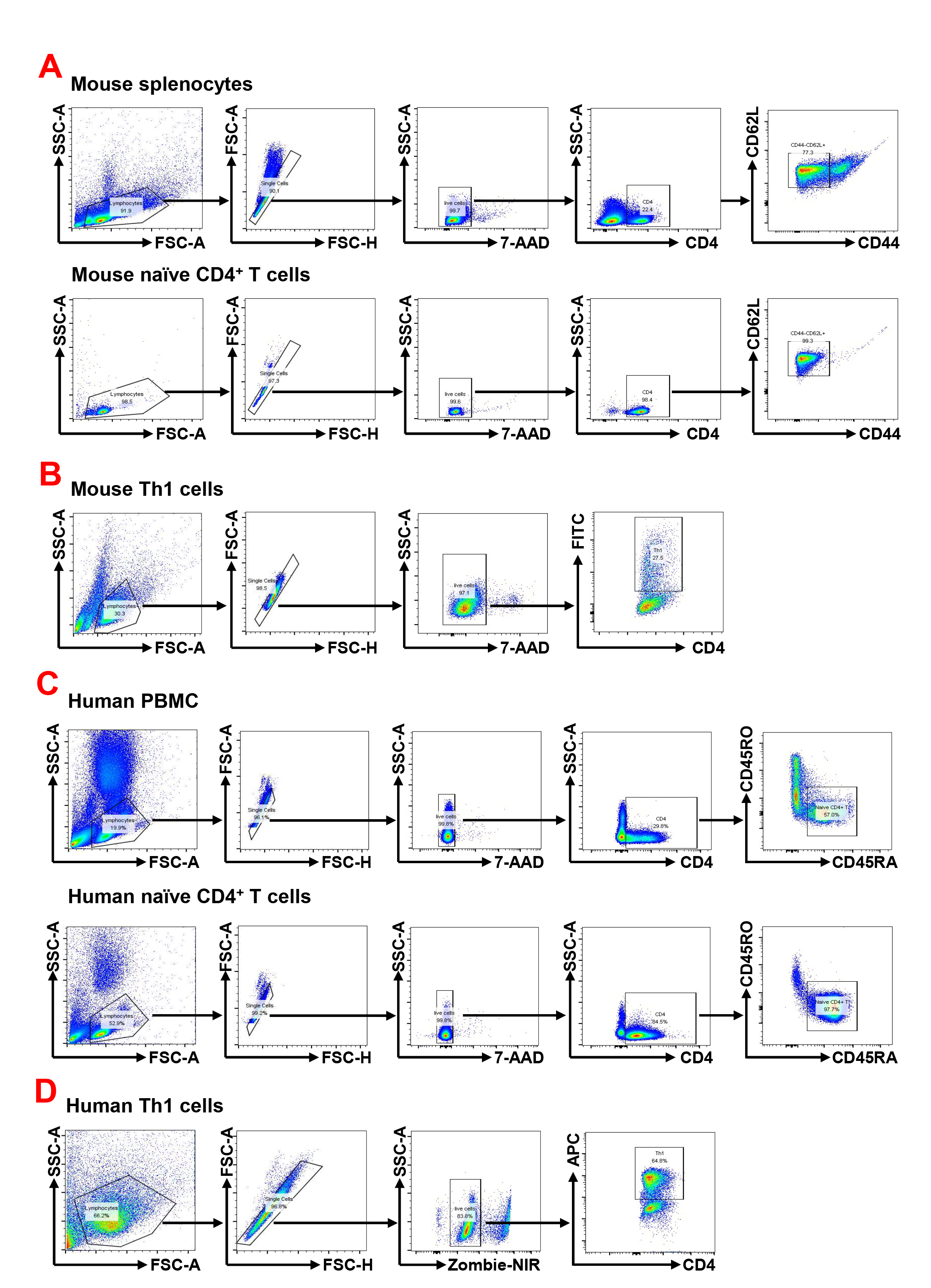

Supplement: Supplementary Figure 2 — Gating strategy for flow cytometry analysis of T cells. (A) Gating of naïve CD4+ T cells in mouse splenocyte (upper panel); the purity of sorted naïve CD4+ T cells (down panel). (B) Gating of Th1 cells in mouse CD4+ T cells. (C) Gating of naïve CD4+ T cells in human PBMC (upper panel); the purity of sorted naïve CD4+ T cells (down panel). (D) Gating of Th1 cells in human CD4+ T cells. PBMC, peripheral blood mononuclear cells. [file Image_2.tif]

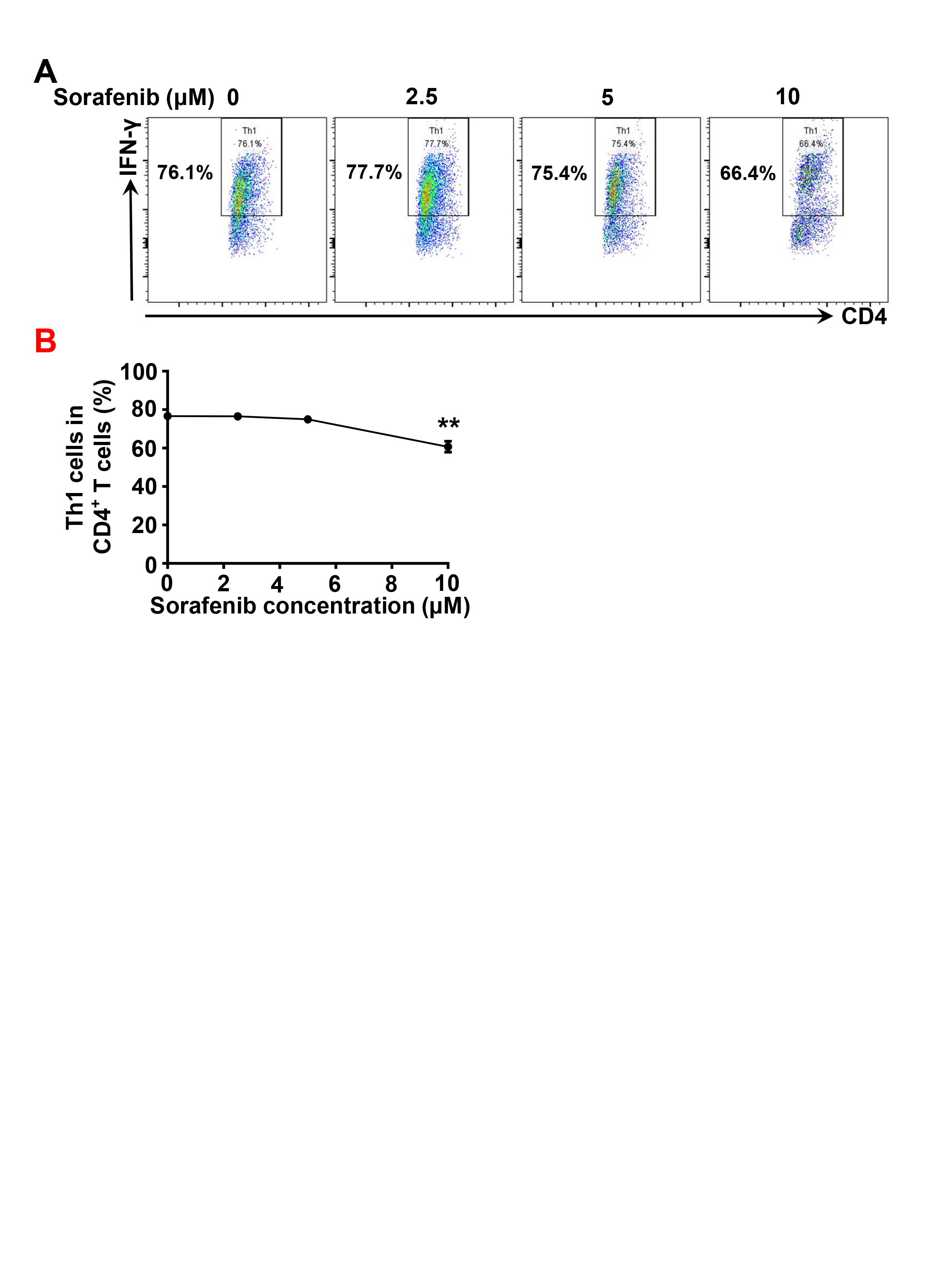

Supplement: Supplementary Figure 3 — Sorafenib does not alter IFN-γ expression in Th1 cell. (A, B) Flow cytometry analysis of IFN-γ + Th1 cells treated with different concentrations of sorafenib (μM) as indicated. [file Image_3.tif]

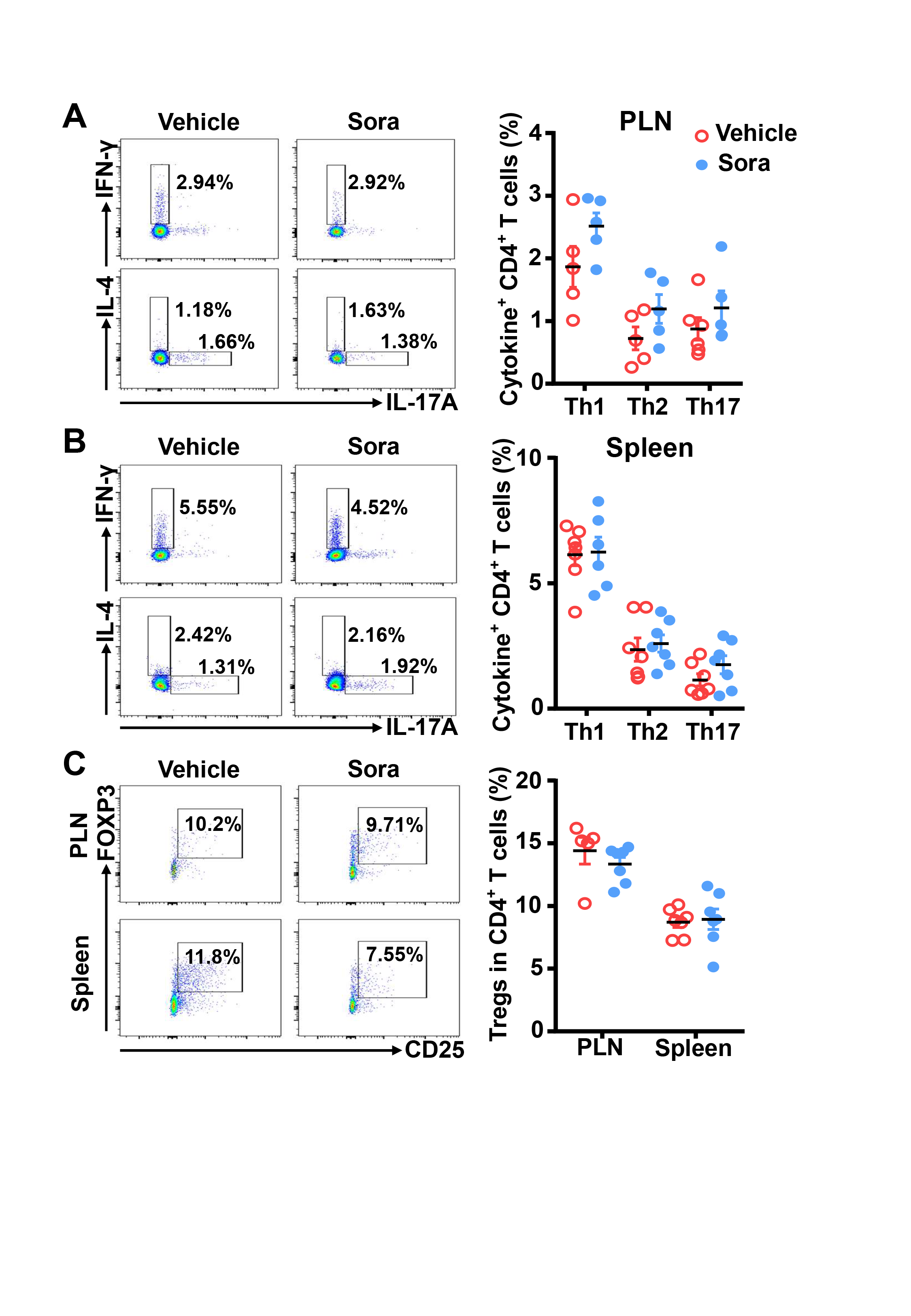

Supplement: Supplementary Figure 4 — Sorafenib does not alter T cell subsets in peripheral immune tissues of prediabetic NOD mice. Flow cytometry analysis of the frequency of Th1, Th2, and Th17 cells in PLN (A) and splenic (B) CD4+ T cells from vehicle-treated or sorafenib-treated NOD mice. (C) Flow cytometry analysis of the frequency of Tregs in PLN and splenic CD4+ T cells, respectively. Sora, sorafenib; PLN, pancreatic lymph nodes. Data represent means ± s.e.m. and data were pooled from 2 or more independent experiments. n = 5-7 mice per group in each experiment. [file Image_4.tif]

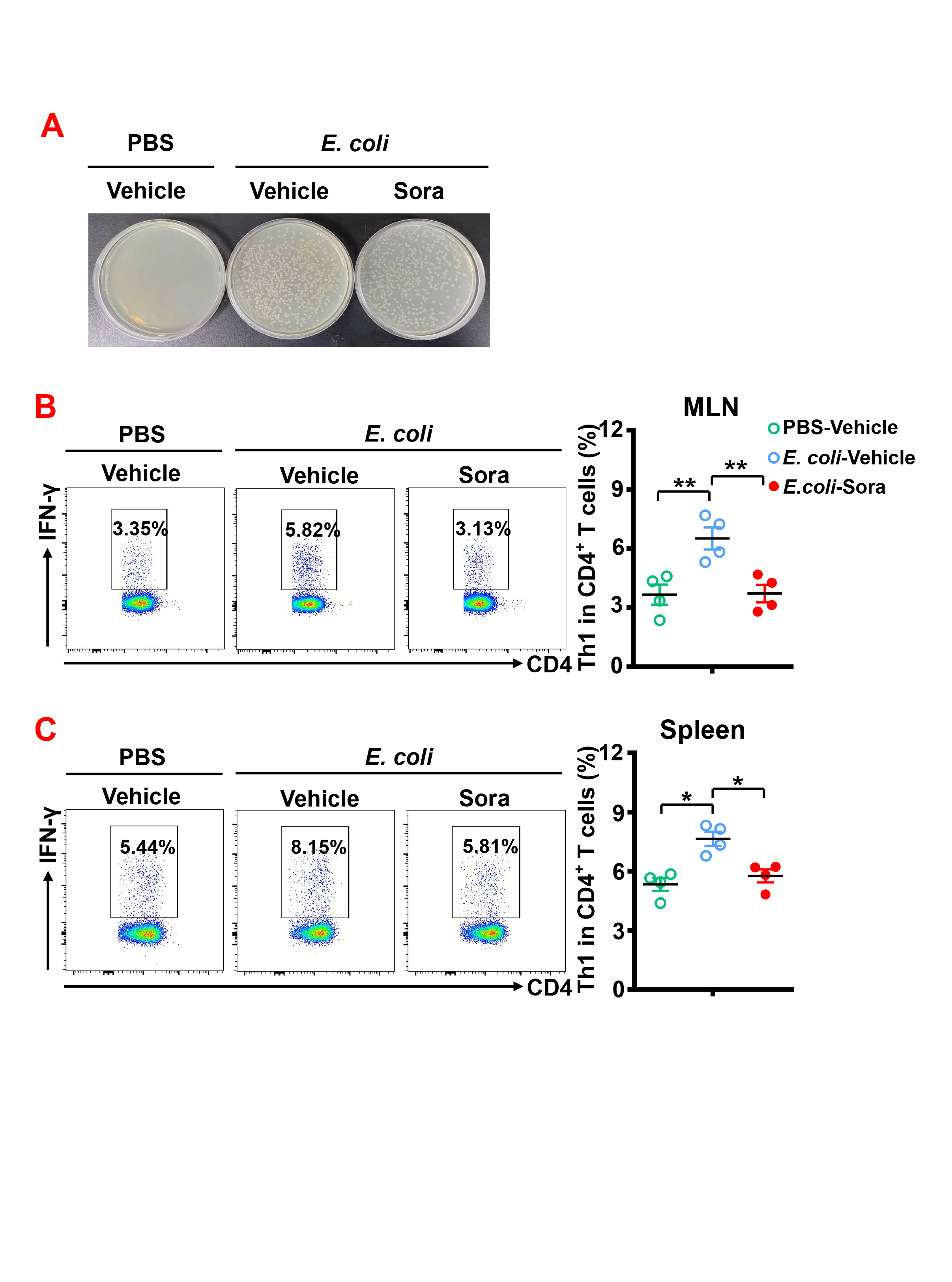

Supplement: Supplementary Figure 5 — Sorafenib decreases activate Th1 cells in MLN and spleen of E. coli-infected mice. (A) Image of E coli on Luria-Bertani Agar plates from vehicle-treated or sorafenib-treated C57BL/6J mice injected with PBS or E.coli (1*107 CFU). Flow cytometry analysis of the frequency of Th1 cells in MLN (B) and splenic (C) CD4+ T cells from the three groups mice as indicated. Sora, sorafenib; MLN, mesenteric lymph node. Data represent means ± s.e.m.. n = 4 mice per group in this experiment. Statistical significance: *P <0.05 or **P <0.01 versus vehicle by t-test. [file Image_5.tif]

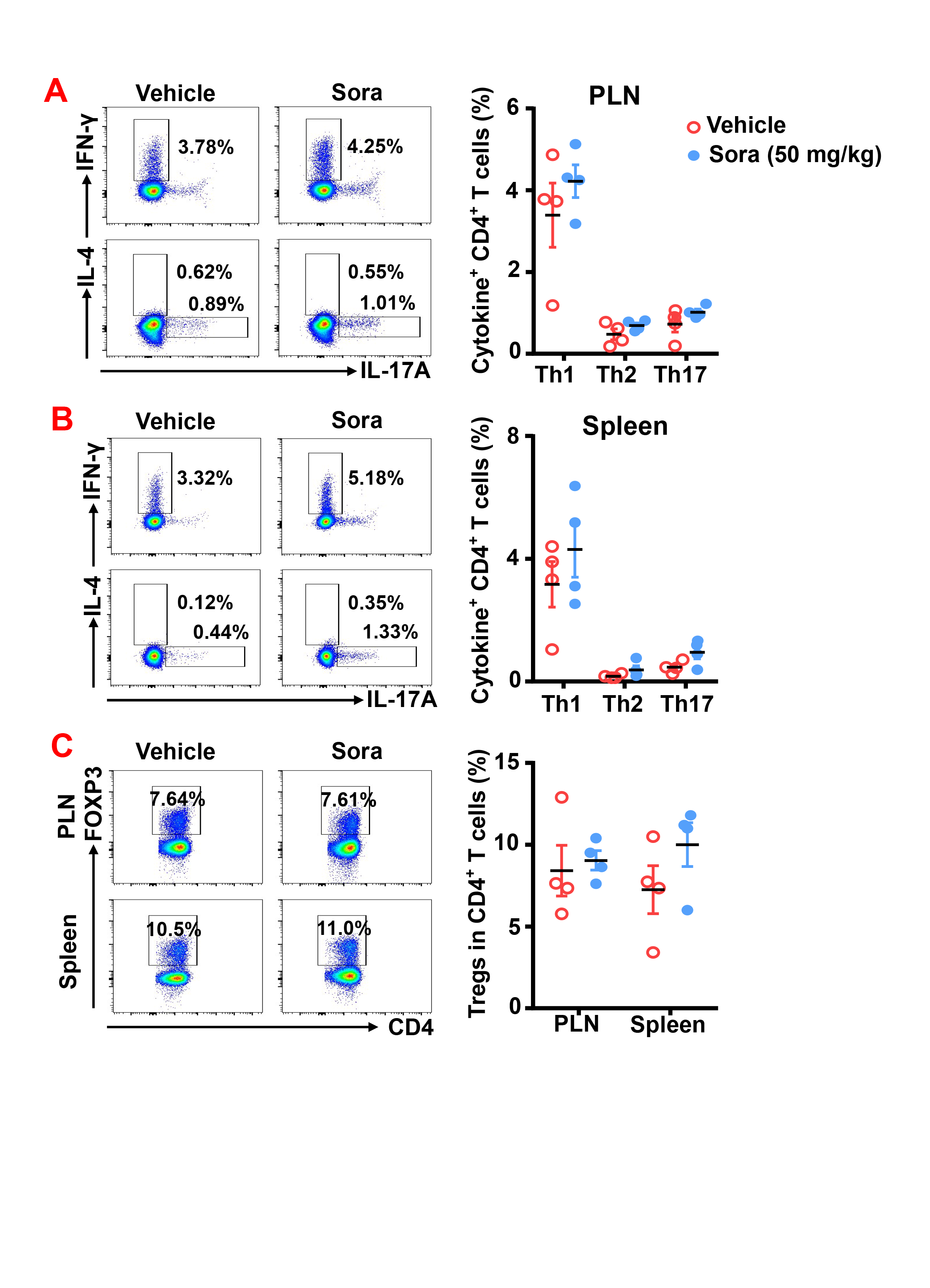

Supplement: Supplementary Figure 6 — Sorafenib does not alter T-cell subsets in peripheral immune tissues of diabetic NOD mice. Flow cytometry analysis of the frequency of Th1, Th2, and Th17 cells in PLN (A) and splenic (B) CD4+ T cells from vehicle-treated or sorafenib-treated diabetic NOD mice. (C) Flow cytometry analysis of the frequency of Tregs in PLN and splenic CD4+ T cells, respectively. Sora, sorafenib; PLN, pancreatic lymph nodes. Data represent means ± s.e.m. n = 4 mice per group in each experiment. [file Image_6.tif]
